# 

**Published:** 1856-04

**Authors:** 


### SUBSCRIBERS TO THE REGISTER.

Those in arrears will find their bills in the present No. of the Register, or will be called on in some other way soon. We hope there will be a prompt response in all cases. We know that it is mere neglect with many that their account runs on from year to year without settling. We have appointed Mr. C. W. James general Collecting agent. His or his agents receipts will in all cases be good.

☞ Mr. C. W. JAMES, No 1 Harrison street, Cincinnati, Ohio, is our General Collecting Agent for the Western States and Texas, assisted by WILLIAM H. THOMAS, THOMAS M. JAMES, Dr. A. L. CHILDS, GEORGE MORRIS, and RICHARD LEEKE. Receipts of either will be good.

### RECEIPTS FOR THE REGISTER.

| | |
|---|---|
| Dr. W. King, vol. 9 | $3 00 |
| Dr. B. A. Satterthwaite, vol. 9 | 3 00 |
| Dr. H. E. Howels | 3 00 |
| Dr. Saml. Griffith | 3 00 |
| Dr.W. H. Goddard, vols. 8 & 9 | 5 00 |
| Dr. E. Bray, vol. 9 | 3 00 |
| Dr. B. Strickland, vol. 9 | 3 00 |
| Dr. J. H. Meredith, vol. 9 | 3 00 |
| Dr. J. G. Hamell, vols. 8 & 9 | 5 00 |
| Dr. McKown, vol. 9 | 3 00 |
| Dr. M. De Camp | 3 00 |
| Dr. E. Hale | 8 00 |
| Dr. D. Parkinson | 7 00 |
| Dr. G. P. Ulrey | 3 00 |
| Dr. E. T. Tibbatts | 3 00 |
| Drs. Duval & Martin | 3 00 |
| Dr. J. Sanford, vols. 9 & 10 | 5 00 |
| Dr. J. B. Newbrough | 1 00 |
| Dr. R. D. Myers | 3 00 |
| Dr. Goodrich, vol. 9 | $3 00 |
| Dr. Lukes | 3 00 |
| Dr. C. L. Stockwell | 3 00 |
| Dr. G. S. Miles | 3 00 |
| Dr. J. R. Reynolds | 3 00 |
| Dr. G. W. Keeley | 3 00 |
| Dr. C. Bonsall | 3 00 |
| Dr. E. Collins | 3 62 |
| Dr. John Harris | 3 00 |
| Dr. N. P. Allen | 3 00 |
| Dr, E. Stone | 3 00 |
| Dr. J. W. Keeley | 3 00 |
| Dr. T. P. Lucas | 4 00 |
| Dr. H. B. Young | 3 00 |
| Dr. F. M. Perry | 3 00 |
| Dr. John Hood | 3 00 |
| Dr. C. W. Kelsey | 3 00 |
| Dr. George Motter | 3 00 |

